# Fabrication and Characterization of High-Frequency Ultrasound Transducers Based on Lead-Free BNT-BT Tape-Casting Thick Film

**DOI:** 10.3390/s18093166

**Published:** 2018-09-19

**Authors:** Junshan Zhang, Wei Ren, Yantao Liu, Xiaoqing Wu, Chunlong Fei, Yi Quan, Qifa Zhou

**Affiliations:** 1 Electronic Materials Research Laboratory, Key Laboratory of the Ministry of Education & International Center for Dielectric Research, School of Electronic and Information Engineering, Xi’an Jiaotong University, Xi’an 710049, Shaanxi, China; jshzhang@stu.xjtu.edu.cn (J.Z.); liuyt8115@stu.xjtu.edu.cn (Y.L.); xqwu@mail.xjtu.edu.cn (X.W.); quanyi@stu.xjtu.edu.cn (Y.Q.); 2 School of Equipment Engineering, Engineering University of People’s Armed Police, Xi’an 710086, Shaanxi, China; 3 School of Microelectronics, Xidian University, Xi’an 740071, Shaanxi, China; clfei@xidian.edu.cn; 4 Department of Ophthalmology and Biomedical Engineering, National Institutes of Health (NIH) Transducer Resource Center, University of Southern California, Los Angeles, CA 90089, USA; qifazhou@usc.edu

**Keywords:** high-frequency ultrasound transducer, lead-free BNT-BT thick film, tape-casting method, pulse-echo method

## Abstract

A lead-free 0.94(Na_0.5_Bi_0.5_) TiO_3_-0.06 BaTiO_3_ (BNT-BT) thick film, with a thickness of 60 μm, has been fabricated using a tape-casting method. The longitudinal piezoelectric constant, clamped dielectric permittivity constant, remnant polarization and coercive field of the BNT-BT thick film were measured to be 150 pC/N, 1928, 13.6 μC/cm^2^, and 33.6 kV/cm, respectively. The electromechanical coupling coefficient *k_t_* was calculated to be 0.55 according to the measured electrical impedance spectrum. A high-frequency plane ultrasound transducer was successfully fabricated using a BNT-BT thick film. The performance of the transducer was characterized and evaluated by the pulse-echo testing and wire phantom imaging operations. The BNT-BT thick film transducer exhibits a center frequency of 34 MHz, a −6 dB bandwidth of 26%, an axial resolution of 77 μm and a lateral resolution of 484 μm. The results suggest that lead-free BNT-BT thick film fabricated by tape-casting method is a promising lead-free candidate for high-frequency ultrasonic transducer applications.

## 1. Introduction

Ultrasound imaging system have been widely used for imaging soft tissues, blood vessels, skin and eyes [1,2,3,4], in which ultrasound transducers transmit ultrasound waves into the tissues and receive the resultant echo waves. Due to the differences in acoustic properties of the tissues, the system can generate an image of the tissues based on the reflected waves. As the key component of ultrasound imaging system, ultrasound transducers ultimately determine the spatial resolution of the imaging system. High frequency and broadband ultrasound transducers have been desired for clinical and biophysical applications [5,6], because they can generate narrow ultrasound beams and have superior spatial resolution [7]. The fabrication of high-frequency ultrasound transducer is strictly reliant on the technology level of the piezoelectric materials and manufacturing techniques. It is well known that the selection of piezoelectric materials has a significant influence on the performance of high-frequency transducers.

For the past several decades, PZT-based (Pb[Zr_x_Ti_1-x_]O_3_, abbreviated as PZT) materials have been commercially applied in the ultrasound imaging field because of their excellent piezoelectric properties [8,9]. However, the element lead (Pb), as a heavy metal, is both harmful to human health and environmentally hazardous. Thus, in recent years, lead-free piezoelectric materials have drawn increasing attention around the world. Among the lead-free piezoelectric materials, bismuth sodium titanate, Na_0.5_Bi_0.5_TiO_3_ (abbreviated as BNT) is one of the most outstanding materials, as it has a high Curie temperature (T_c_ = 320 °C) and stable electrical properties [10]. According to the literature, bulk BNT-based ceramics have been applied in various applications such as high-frequency ultrasound transducers for medical applications, piezoelectric actuators and ultrasound transducers for nondestructive evaluation [11,12,13].

To enhance the electrical properties of BNT-based polycrystalline ceramics, (Bi_0.5_Na_0.5_) TiO_3_- x BaTiO_3_ (abbreviated as BNT-BT) binary system piezoelectric ceramics have been thoroughly studied for years. The research indicates the ceramics transform from rhombohedral phase to tetragonal phase, and the morphotropic phase boundary (MPB) exists at x = 0.06–0.07; and thus the ceramics exhibit the optimized piezoelectric characteristics.

In traditional fabrication procedures of high-frequency transducers, it is challenging and time-consuming to lap down the bulk piezoelectric materials, including piezoelectric ceramics or crystals, to the order of tens of microns. Therefore, piezoelectric thick films are low-cost alternatives and the sol-gel and tape-casting technology is convenient for high frequency transducer fabrication.

In this paper, lead-free BNT thick film doped with BaTiO_3_ was prepared using the sol-gel and tape-casting method, and the structural and electrical properties of the BNT-BT thick film were evaluated. A high-frequency ultrasound transducer was fabricated using the BNT-BT thick film. The performance of this ultrasound transducer was investigated in detail.

## 2. Materials and Methods

First, 0.94 (Na_0.5_Bi_0.5_) TiO_3_-0.06BaTiO_3_ thick film (abbreviated as BNT-BT thick film) was prepared using –Bi_2_O_3_ (≥99.8%), Na_2_CO_3_ (≥99.8%), BaTiO_3_ (≥99.5%), TiO_2_ (≥98.0%) analytical-grade powders (Sinopharm Chemical Reagent Co., Ltd., Shanghai, China) as raw materials using the tape-casting method. The slurry for casting was prepared by a series of procedures, which were, in sequence: 7 h ball milling, calcining at 900 °C for 2 h, grinding, addition of solution and dispersant, and bubble defoaming. Then, the prepared composite slurry was cast on a polyethylene terephthalate (PET) film using a casting machine (LYJ-150, Dongfang Taiyang Company, Beijing China). The cast samples were then processed for several steps: First, the samples were left for 24 h at 25 °C for the evaporation of organic solvent; next, the samples were pre-heated at 600 °C for 2 h to remove organic components; and last, the samples were sintered at 1160 °C for 2 h in a high temperature furnace. This produced a lead-free BNT-BT thick film. Cr/Au (50 nm/100 nm) electrodes were deposited on both sides of the BNT-BT thick film using magnetron sputtering technique for characterization measurement.

Surface morphology of the BNT-BT thick film was identified by a field-emission scanning electron microscope (FESEM, Quanta 250 FEG, FEI, Hillsboro, OR, USA). The phase structure of the thick film was characterized using an X-ray diffraction meter (XRD, D/MAX-2400, Rigaku, Tokyo, Japan). The polarization-electric hysteresis loop of the sample was measured using a ferroelectric testing system (TF Analyzer 2000, aixACCT, Aachen, Germany) at 100 Hz. A DC electric field of 4.2 × 10^6^ V/m was applied on the thick film for 60 min, and the poling was executed in a silicone oil bath at 80 °C. The poled samples were aged for a minimum of 72 h at room temperature before any electrical measurements were taken. The longitudinal piezoelectric coefficient d_33_ was measured using a quasi-static d_33_ testing device (ZJ-1, CAS, Beijing, China). The electrical impedance characteristics of the BNT-BT thick film were measured using the resonance method by an electric impedance analyzer (HP 4294A, Agilent Technologies, Santa Clara, CA, USA).

**Figure 1:**
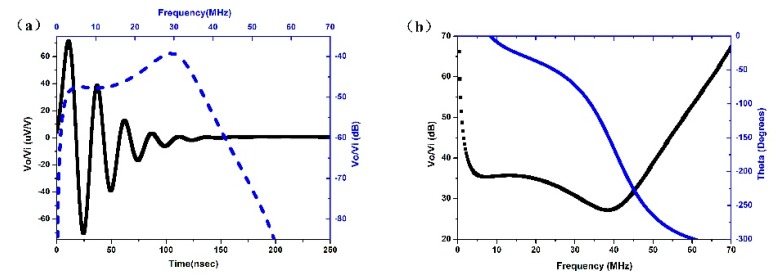
Modeled performances of transducers based on BNT-BT thick film: (**a**) the pulse-echo wave and frequency spectrum; (**b**) the two-way insertion loss.

The transducer was designed using the KLM model-based simulation software Piezo CAD (Sonic Concepts, Woodinville, WA, USA), shown in Figure 1. The simulation parameters were set as, thickness of 60 μm, element area size of 1 mm × 1 mm, and clamped dielectric constant of 1920. The simulation results indicated the −6 dB center frequency was 35 MHz, bandwidth was 38.9%, and the two-way insertion loss was about 27 dB.

**Figure 2:**
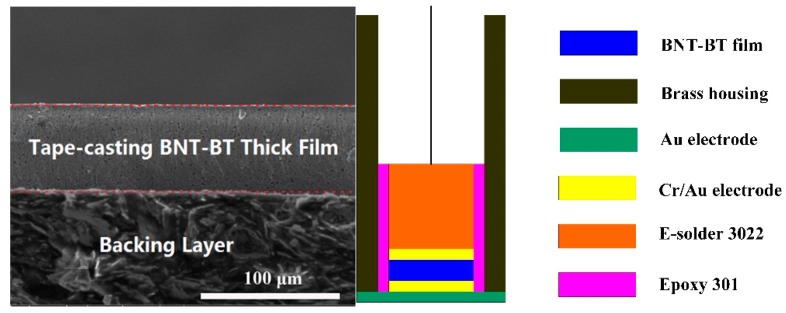
SEM section image and schematic diagram of the BNT-BT transducer.

The transducer fabrication was carried out by the traditional method illustrated in the literature [14,15]. First, Cr/Au (50 nm/100 nm) electrodes were sputtered on both sides of the BNT-BT thick film, and an E-solder 3022 (Von Roll Isola Inc., New Haven, CT, USA) layer with an acoustic impedance of 5.92 MRayls was cured on one side by centrifuged casting as backing layer. The SEM of sample cross-section and the BNT-BT transducer schematic diagram are illustrated in Figure 2.

**Figure 3:**
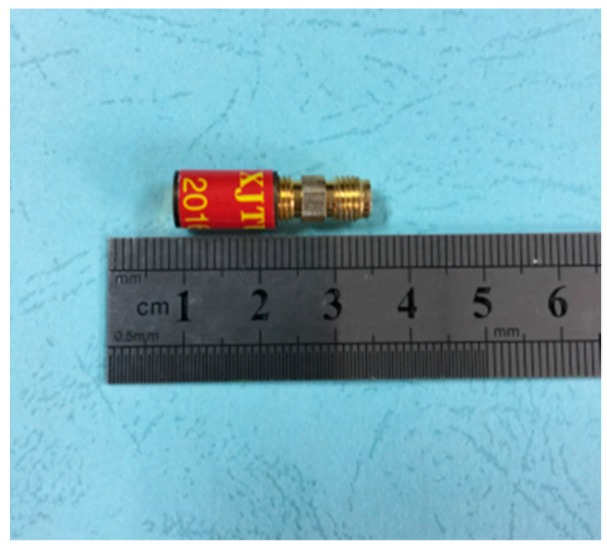
Photograph of the BNT-BT transducer.

Next, the sample was back-lapped to the level of 600 μm thick, and mechanically diced into square posts with area dimensions of 1 mm × 1 mm. Then, a copper wire was welded to the backing layer of the post using conductive epoxy, and the whole post was fixed into a brass housing with SMA connector. Last, Au layer (100 nm) was sputtered across the housing to form a ground electrode. The photograph of the BNT-BT transducer is shown in Figure 3.

The performance of the BNT-BT transducer was evaluated by a pulse-echo mode operation in de-ionized water tank at room temperature. The transducer was excited by a dual pulser/receiver (JSR Ultrasonics DPR 500, Imaginant Inc., Pittsford, NY, USA) with 200 Hz pulse repetition frequency and 50-Ω damping. The output gain control was adjusted to 14 dB to obtain a signal level between a ±0.2 and ±0.5 V peak. An X-cut quartz crystal block was used as an ultrasound waveform reflecting target and the location of the block was at the focal surface of the transducer.

The reflected waveform was received and digitized by an oscilloscope (InfiniiVision DSO-X 3104A, Keysight Technologies, Inc., Colorado Springs, CO, USA) with 50-Ohm coupling. The frequency spectrum of the received waveform was analyzed by the Fast Fourier Transform (FFT) operation. The center frequency (*f_c_*) and the −6 dB fractional bandwidth (BW) could be determined by the following equations:

(1) $$f_{c}=\frac{f_{l}+f_{u}}{2}\;$$

(2) $$BW=\left(\frac{f_{u}-f_{l}}{f_{c}}\right)\times 100\%\;$$

where *f_l_* and *f_u_* are the two −6 dB points of the power spectrum, defined as lower and upper band edges of frequency, respectively, at which the magnitude of the amplitude in the spectrum is 50% (−6 dB) of the maximum.

The −6 dB axial and lateral resolutions of the BNT-BT transducer were also measured by wire target imaging. The test was carried on in a deionized water tank at room temperature, in which both the BNT-BT transducer and a tungsten wire target were placed. The Panamatrics 5900PR was used to excite the transducer, and a 12-bit data acquisition card (Gage 12400, Gage Applied Technologies, Lockport, IL, USA) was used to fulfill the analog to digital data conversion and data transfer to computer, working at the sampling frequency up to 400 MHz. The tungsten wire targets were scanned across the BNT-BT transducer sound field with a step size of 5 μm. The amplified echo signals were acquired at each step by the Gage 12,400 and then the data were used to form an ultrasound wire phantom image offline. The −6 dB spatial resolutions of the transducer were then measured from the axial and lateral line spread functions of the recorded wire image.

## 3. Results

### 3.1. BNT-BT Thick Film Charaterization

**Figure 4:**
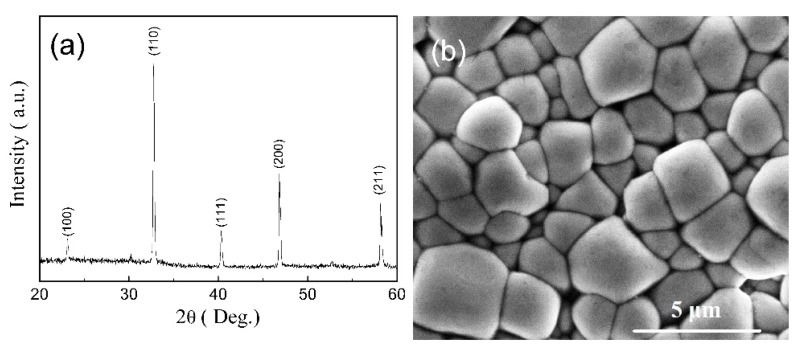
XRD pattern (**a**) and SEM surface image (**b**) of the BNT-BT thick film.

Figure 4 shows the XRD pattern and SEM image of the BNT-BT thick film. From Figure 4a, we can see that the BNT-BT thick film has a single perovskite structure without secondary phase detected, indicating the formation of a solid solution of BNT and BT. Figure 4b shows that the BNT-BT thick film exhibits a dense and uniform structure.

**Figure 5:**
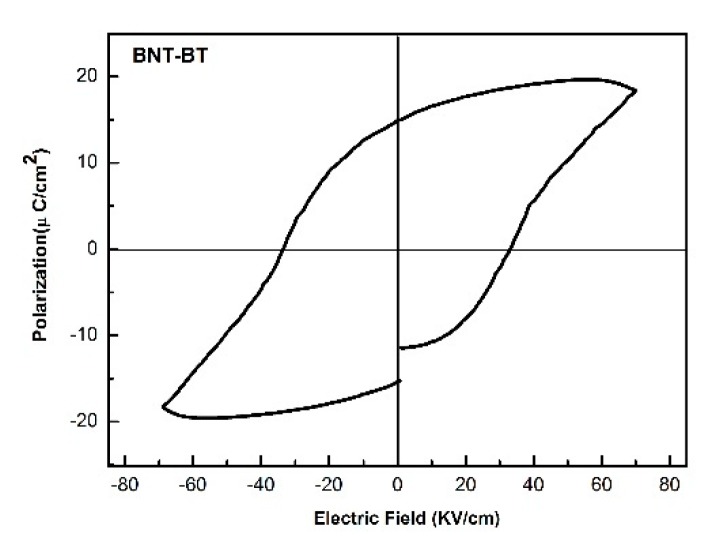
The P-E hysteresis loop of BNT-BT thick film.

The P-E hysteresis loop of BNT-BT thick film is presented in Figure 5, which indicates that the hysteresis loop is well saturated, and the remnant polarization P_r_ and coercive filed E_c_ are 13.6 μC/cm^2^ and 33.6 kV/cm, respectively.

**Figure 6:**
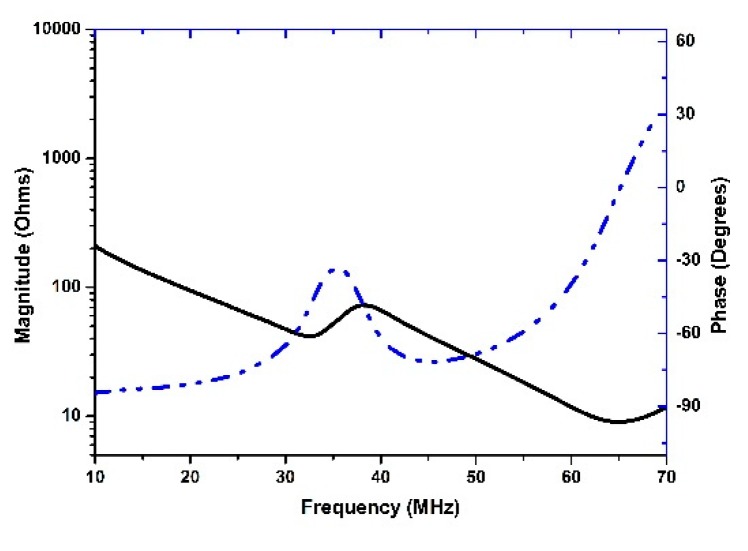
Measured electrical impedance magnitude (solid line) and phase angle (dash line) of BNT-BT tape-casting thick film.

The piezoelectric coefficient d_33_ is found to be around 150 pC/N. The electrical impedance characteristics are displayed in Figure 6, showing that resonant and anti-resonant peaks are located at 32.5 MHz and 37.9 MHz, respectively.

According to the IEEE standard [16], the longitudinal electromechanical coupling coefficient *k_t_* is given by Equation (3):

(3) $$k_{t}=\sqrt{\frac{\pi }{2}\frac{f_{r}}{f_{a}}\tan\left(\frac{\pi }{2}\frac{f_{a}-f_{r}}{f_{a}}\right)}\;$$

where *f_r_* and *f_p_* are series and parallel resonate frequencies. The *k_t_* value is calculated to be 0.55, and it is a relatively high value among the reported lead-free thick films [17]. Additionally, the phase curve of the thick film has a peak at 35.2 MHz, which indicates that the center frequency of the BNT-BT thick film transducer is nearby.

### 3.2. BNT-BT Transducer Characterization

**Figure 7:**
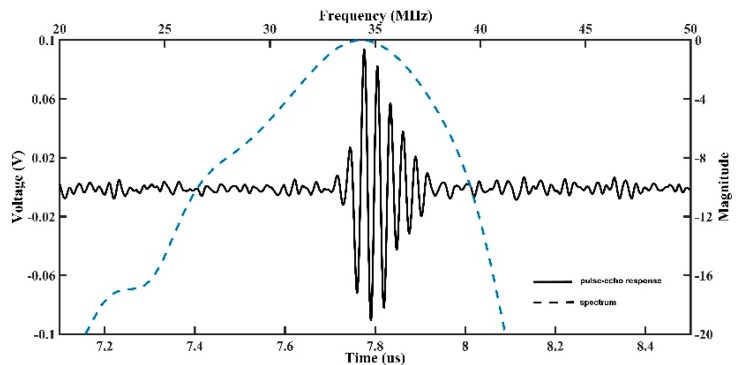
Measured pulse-echo response and Fast Fourier Transform (FFT) spectrum of the BNT-BT transducer.

The measured pulse-echo waveform and frequency spectrum of the BNT-BT transducer are shown in Figure 7. The *f_l_* and *f_u_* were found to be 29.6 and 38.4 MHz, respectively. According to Equations (1) and (2), the center frequency of the BNT-BT transducer was 34 MHz, and the fractional bandwidth at −6 dB was about 26%. The relatively low fractional band width may be caused by the fact that the BNT-BT transducer was measured without a matching layer. Further improvement would be done in the future. Without signal amplification, the amplitude of the pulse-echo waveform is approximately 180 mV.

The two-way insertion loss (IL) was calculated from the ratio of the received pulse spectrum amplitude to the source spectrum. After the signal loss from transmission into the quartz target (1.8 dB) and the attenuation in water (2.2 × 10^−4^ dB (mm·MHz^2^)^−1^) was compensated for, the insertion loss is described in dB by Equation (4):

(4) $$IL=20\log\frac{V_{R}}{V_{T}}+1.8+2.2\times 10^{-4}\times 2d\times f_{c}^{2}\;$$

where *V_T_* and *V_R_* are the amplitudes of transmitting and receiving waveforms, respectively, in volt; d is the distance between the transducer and the crystal block in millimeters; *f_c_* is the transducer center frequency in Mega Hertz. The minimum insertion loss of the BNT-BT transducer was calculated to be −29 dB.

**Figure 8:**
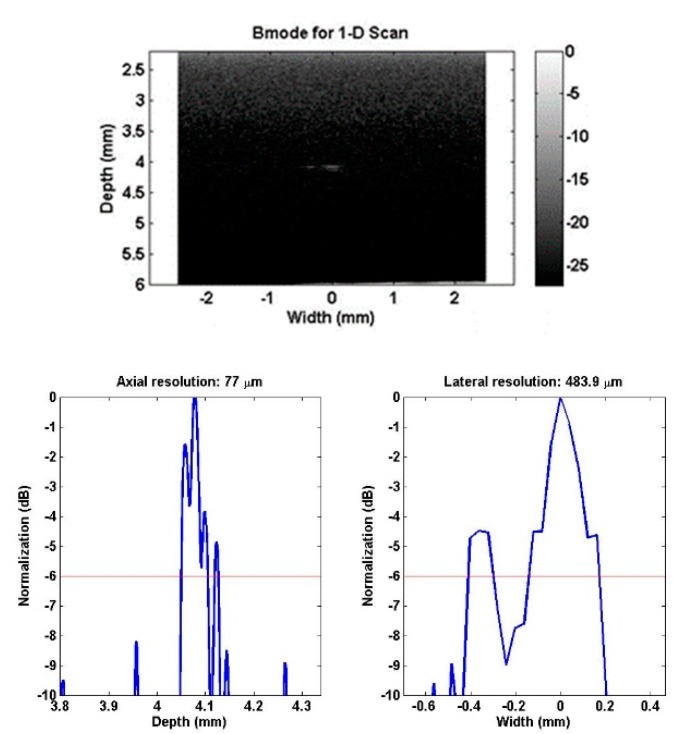
Wire target image and −6 dB resolutions of BNT-BT transducer.

The measured −6 dB resolutions, also named FWHM (full-width half-maximum) resolutions, were 77 μm and 484 μm for the axial and lateral directions, respectively, as illustrated in Figure 8.

The axial resolution can be calculated according to Equation (5),

(5) $$R_{axial}=\frac{c\cdot T_{pulse}}{2}=\frac{c}{2\cdot B_{pulse}}\;$$

Using the −6 dB pulse length of 0.093 μs and the acoustic velocity of 1450 m/s, the axial resolution can be calculated to be 67 μm, which is close to the measured value of 77 μm. The lateral resolution is mainly determined by the width of the ultrasound beam. The BNT-BT thick film transducer was not focused, and this may be the cause of the relatively low lateral resolution.

### 3.3. Ultrasound Bio-Microscope Imaging

**Figure 9:**
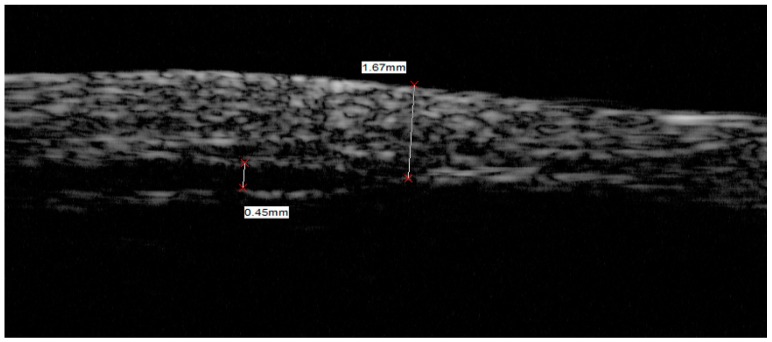
Image of human skin from the back of the hand, generated by BNT-BT transducer using Ultrasound Bio-Microscope (UBM).

The preliminary imaging test was carried out by an ultrasound bio-microscope (SW 3200L, Suoer, Tianjin, China) and the dermatologic image captured is presented in Figure 9. The image from the skin of the back of a human hand was captured by the fabricated BNT-BT transducer without a matching layer. The imaging process was carried on in a deionized water bath, with the transducer scanning close and vertical to the skin surface. The layered structure can be distinguished in the image as the epidermis, dermis and subcutaneous tissue, in which the edge of the epidermis and dermis is not clear enough. The image captured by the BNT-BT transducer has relatively large sparkles indicating a relatively low resolution. The bandwidth of the BNT-BT transducer without a matching layer was 26%, so the imaging resolution was not very high. The BNT-BT transducer was not spherically focused and this also caused image quality decay.

This trial showed that the BNT-BT transducer can be used in ultrasound imaging, and further improvement should be carried on to enhance the lead-free transducer performance with higher frequency which may be used in skin imaging.

## 4. Discussion

In this work, we utilized the piezoelectric tape-casting thick film technique for a high-frequency lead-free ultrasound transducer. The utilization of BNT-BT tape-casting thick film may avoid and simplify the lapping procedures currently used in the fabrication of high-frequency ultrasound transducers. A high frequency ultrasound transducer (35 MHz) was fabricated and tested using the lead-free piezoelectric thick film. The testing results indicated that the BNT-BT transducer exhibited relatively high sensitivity and resolution. However, the capabilities in regards to skin imaging still need to be enhanced. An increase in frequency will allow the resolution and contrast to be further improved, which can be achieved using the tape-casting piezoelectric thick film. A spherical focused transducer will also lead to higher imaging resolution and this could be easily realized.

## 5. Conclusions

In this paper, a high frequency ultrasound transducer was fabricated successfully based on a lead-free BNT-BT tape-casting thick film. The pulse-echo response performances showed that the BNT-BT thick film transducer had a center frequency of 34 MHz, and a fractional band width of 26%. The measured −6 dB resolutions estimated from the line spread functions were 77 μm in the axial direction and 484 μm in the lateral direction. The preliminary skin imaging test was tried using the BNT-BT transducer and an image of skin from the back of the hand was successfully captured. The experiment results suggest that the lead-free piezoelectric BNT-BT tape-casting thick film may be a promising candidate material for high frequency ultrasound transducer applications.
